# Efficacy and safety of generic imatinib after switching from original imatinib in patients treated for chronic myeloid leukemia in the United States

**DOI:** 10.1002/cam4.2545

**Published:** 2019-09-10

**Authors:** Iman Abou Dalle, Hagop Kantarjian, Jan Burger, Zeev Estrov, Maro Ohanian, Srdan Verstovsek, Farhad Ravandi, Gautam Borthakur, Guillermo Garcia‐Manero, Elias Jabbour, Jorge Cortes

**Affiliations:** ^1^ Department of leukemia MD Anderson Cancer Center The University of Texas Houston Texas

**Keywords:** bioequivalence, generic, imatinib, leukemia, safety

## Abstract

**Introduction:**

Imatinib is standard therapy for patients with chronic myeloid leukemia (CML). In February 2016, a generic formulation entered the US market. Physicians and patients are frequently concerned about whether switching from original to generic drugs may affect the efficacy and/or safety.

**Materials and methods:**

This is an observational retrospective study using medical charts of patients diagnosed with CML in the chronic phase who were treated with original imatinib from the year 2000 to 2017 and who were subsequently switched to generic imatinib.

**Results:**

In this study, 38 patients have switched to generic imatinib. Before the switch, responses were assessed on all patients, all of them were in CCyR and 36 (95%) were in MMR, including 28 (74%) with MR4.5. Patients have received generic imatinib for a median of 19.4 (range, 3.4‐46.3) months. Molecular responses after switching were stable in 89%, improved in 8%, and worsened in 3% of patients. After switching, 15 (39%) patients reported new or worsening adverse events, including 5 (13%) patients with edema, 8 (21%) muscle cramps, 7 (18%) nausea, 6 (16%) diarrhea, and 5 (13%) fatigue.

**Discussion:**

Bioequivalence studies demonstrated the same rate and extent of absorption of generic imatinib compared to the original form, which led to the FDA approval. In our observational series, most of the patients maintained their responses and none lost MMR. Adverse events noted were mild and well tolerated.

**Conclusion:**

A change from original to generic imatinib appears to maintain efficacy and be generally safe. More patients and longer follow‐up are required to confirm these observations.

## Graphical abstract

Generic imatinib entered the US market in February 2016. In this observational retrospective study, patients treated for chronic myeloid leukemia who switched from original to generic form of imatinib maintained same responses in 89% of patients and had no reported major adverse events.

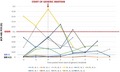

## 1 INTRODUCTION

Imatinib, an oral first‐generation inhibitor of the BCR‐ABL1 tyrosine kinase, was a game‐changing discovery in the treatment of patients with chronic myeloid leukemia (CML).^1^ In May 2001, the US Food and Drug Administration (FDA) approved imatinib (Novartis pharmaceuticals—Glivec, Gleevec) in relapsed and refractory CML after interferon‐based therapy.^2^ One year later, this indication expanded to patients with newly diagnosed CML.^3^ Patients with CML treated with imatinib have a significant improvement of survival time approaching that of the general population, with a 10‐year overall survival of 83.3% reported on the latest update of the pivotal IRIS trial.^4^, ^5^, ^6^ Imatinib first became available in its generic form in the United States in February 2016.^7^ Sun Pharmaceuticals from India manufactured the first generic form of imatinib marketed in the United States. After expiration of its market exclusivity, several other generic forms from different manufacturers (Apotex, Teva, and Mylan) have entered the market. The use of generic drugs, if priced at considerably lower levels than the brand form, may help reduce the financial burden on patients and healthcare systems.^8^, ^9^ Since financial consideration is one of the factors cited by patients to explain decreased adherence and desire for treatment discontinuation, a price reduction could potentially increase patient's adherence, an important pre‐requisite for improved long‐term outcomes.^10^ However, patients and physicians are frequently concerned with the use of generic formulations of antineoplastic agents due to lack of large comparative studies evaluating the efficacy and safety of the generic formulations with those obtained with original formulations.^11^, ^12^, ^13^ Generic forms of imatinib were approved in the European Union in 2012 and Canada in 2013. They were also marketed in many developing countries years before the original patent expiration. Many reports have been published, mainly from Egypt, Morocco, Iran, and Iraq, reporting lack of efficacy of generic form of imatinib, including loss of hematologic response.^14^, ^15^, ^16^, ^17^, ^18^, ^19^ These reports frequently include unregulated formulations of generic agents and copies with poor quality controls. The lack of comparative studies and the emergence of reports questioning the efficacy of the generic form of imatinib raised many concerns among physicians and patients. To address this concern, we analyzed the efficacy and safety of the generic form of imatinib approved by the FDA in adult patients diagnosed with CML in the chronic phase.

## 2 METHODS

### 2.1 Study design and participants

This is a retrospective, observational, chart review study including adult patients diagnosed with CML in the chronic phase, treated and followed at the University of Texas MD Anderson Cancer Center. Patients treated with original imatinib either as frontline therapy or after interferon therapy from January 2000 until December 2017 and subsequently switched to a generic form of imatinib approved by the FDA are included in the analysis. The switch was mandated by the insurance company and not the physician's choice. The last follow‐up date was in February 2019.

The study was performed in accordance with the Declaration of Helsinki. The Institutional Review Board approved the collection of data and a waiver of informed consent was granted for this chart review study.

### 2.2 Procedures, objectives, and endpoints

Response assessment before the switch was performed in all patients as per standard practice using standard G‐banding technique for cytogenetic analysis and/or peripheral blood fluorescent in situ hybridization (FISH) to assess cytogenetic response, and *BCR‐ABL* RT‐PCR to assess molecular response. Cytogenetic responses were classified by standard criteria and included complete cytogenetic response (CCyR) (0% Ph‐positive metaphases), partial cytogenetic response (PCyR) (1%‐35% Ph‐positive metaphases), major cytogenetic response (MCyR) (≤35% Ph‐positive metaphases), and minor cytogenetic response (>35% to 95% Ph‐positive metaphases). A major molecular response (MMR) was defined as *BCR‐ABL1/ABL1* transcript ratio ≤0.1% on international scale (IS), and MR4.5 as a ratio of ≤0.0032% IS.

After switching to generic imatinib, the frequency of complete blood counts, creatinine level, liver function tests, and lactate dehydrogenase blood checks was increased to every 1‐2 months, and *BCR‐ABL* RT‐PCR testing was performed on peripheral blood 3 months after the switch regardless of baseline response, and every 6 months thereafter. Any rise in transcript levels prompted more frequent monitoring. A confirmed loss of MMR (i.e, documented at two consecutive time points) was considered as a failure to maintain the same efficacy as original imatinib.

Patients were specifically assessed for new or worsened adverse events in subsequent visits after treatment change. Patients were interviewed and examined during clinic visits at our institution and medical records for visits to other institutions were reviewed for any new or aggravating subjective or objective adverse events. All laboratory values at our institution and other institutions during the observation period were also reviewed. Adverse events were graded according to the National Cancer Institute Common Terminology Criteria for Adverse Events, version 4.0.

Descriptive statistics including mean, median, and range for continuous variables such as laboratory measurements, frequency counts, and percentages for categorical variables such response status are provided.

## 3 RESULTS

### 3.1 Patient characteristics

A total of 38 patients have switched from original to generic imatinib. Baseline characteristics are summarized in Table 1. Most (66%) of the patients were receiving imatinib at a dose of 400 mg prior to switching. Most patients continued the same dose of imatinib upon switching except for one patient who had an increase in the dose of imatinib from 400 mg to 600 mg at the time of switching due to inadequate response. In this study, 29 (76%) patients were receiving imatinib as a frontline treatment for CML, whereas 9 (24%) patients had received interferon therapy prior to imatinib. Responses were assessed in all patients prior to switching to generic imatinib. All patients were in CCyR and 36 (95%) were in MMR, including 28 (74%) with MR4.5. The median duration of original imatinib use prior to the switch to generic was 12 (range, 1.5‐17) years and the median duration in MMR while on original imatinib was 10.6 (range, 0.5‐16.3) years.

**Table 1:** Baseline characteristics

N = 38	n (%), or median [range]
Age at diagnosis, y	40 [10‐66]
Male sex	16 (42)
Imatinib 1st line	29 (76)
Imatinib 2nd line	9 (24)
Imatinib dose, mg	
100	1 (3)
200	2 (5)
300	4 (10)
400	25 (66)
600	5 (13)
800	1 (3)
Time on original imatinib, y	12 [1.5‐17]
Time to achieve CCyR, mo	6.2 [1.2‐29.2]
Time to achieve MMR, mo	11.7 [2.7‐67.7]
Time on generic imatinib, mon	19.4 [3.4‐46.3]

### 3.2 Efficacy

**Figure 1:**
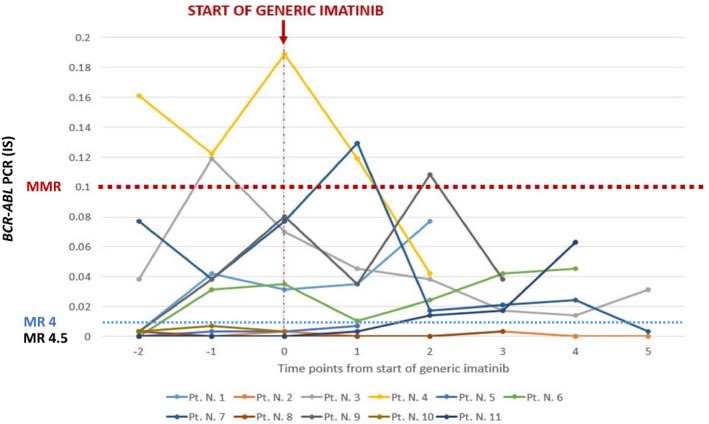
Changes in BCR‐ABL1/ABL1 PCR at multiple time points before and after switching from original to generic imatinib. Only patients with detectable PCR at any time were included in this graph (N = 11). Among patients with undetectable PCR, 27/28 patients maintained MR4.5 after the switch after a median follow‐up of 20 mo

At the time of this analysis, patients have received generic imatinib for a median of 19.4 (range, 3.4‐46.3) months. All patients have maintained CCyR. Among the patients with detectable *BCR‐ABL* PCR before the switch (n = 10), one patient decreased transcripts from 0.07 to 0.0315 IS, one achieved MR4 (transcripts decreased from 0.12 to 0.042 IS), one attained MR 4.5, and seven patients had stable molecular responses (Figure 1). One patient lost MR4.5, but not MMR after switching to generic imatinib and having a dose reduction because of adverse events (mainly fatigue and diarrhea). None of the patients lost MMR. Among patients with MR4.5 at the time of switching, 27 (96%) maintained such response (in all instances with an undetectable *BCR‐ABL* transcript level) while on generic imatinib. Notably, among the eight patients who had MMR but not MR4.5, four had some improvement in the transcript levels after the switch, including one that achieved MR4.5. All patients are alive and none has transformed to the accelerated or blast phase. Five patients electively discontinued generic imatinib after a median duration of MR4.5 of 118 months (range, 102‐181 months), three patients had a progressive increase in their *BCR‐ABL* PCR, including two who lost MMR, and the other two patients remained in MR4.5 after 6 and 24 months of discontinuation, respectively. Of the two patients who lost MMR after elective discontinuation, one resumed imatinib with short follow‐up for response assessment, and the other did not resume therapy while trying to conceive.

### 3.3 Safety

Of all patients, 36 (95%) had experienced at least one adverse event anytime while on original imatinib, mostly muscle cramps in 22 (58%) patients, peripheral edema/periorbital edema in 21 (55%), diarrhea in 14 (37%), fatigue in 13 (34%) and nausea in 11 (29%) patients. Laboratory abnormalities identified were anemia in 13 patients (34%), thrombocytopenia in 3 (8%), neutropenia in 1 (3%), increased creatinine in 5 (13%), and increased bilirubin in 2 (3%) patients. None of the adverse events was grade 3 or 4. After switching to generic imatinib, 15 (39%) patients reported new or worsening adverse events including 5 (13%) patients with edema (including one with new onset periorbital edema), 8 (21%) muscle cramps (including 5 with new onset muscle cramps), 7 (18%) nausea (including 4 with new onset nausea), 6 (16%) diarrhea (two of them with new onset diarrhea), and 5 (13%) fatigue (including 4 with new onset fatigue). New grade 1 anemia occurred in 2 (5%) patients, and an increase in creatinine levels was observed in 4 (11%) patients (from 1.12 to 1.43, 1.1 to 1.32, 0.63 to 1.37 and 1.6 to 3.75 mg/dL, respectively) (Table 2). Of the four patients with an increase in creatinine levels, two switched to second‐generation TKI, one discontinued generic imatinib after meeting criteria for treatment discontinuation and maintained undetectable transcripts after 24 months of follow‐up, and one switched back to innovator imatinib. After such interventions, three patients had normalization of kidney function and one patient had stabilization of creatinine level at 3.7 mg/dL. Two patients had a dose reduction because of adverse events, the first patient had the dose of generic imatinib reduced from 400 to 300 mg/d due to fatigue and diarrhea 2 months after switching from original imatinib, the other patient had dose reduced from 300 to 200 mg/d due to nausea and diarrhea. The symptoms improved upon dose reduction in both patients. One patient skipped four doses because of worsening periorbital edema, then resumed it with intermittent mild edema. Ten patients discontinued treatment with generic imatinib: five had elective discontinuation because of sustained MR4.5, three patients were changed back to original imatinib because of diarrhea, and two patients switched to second‐generation TKI because of renal insufficiency. The first patient was on 200 mg of original imatinib because of creatinine elevation to 1.4 mg/dL that subsequently resolved. After switching to generic, creatinine level increased again gradually reaching 1.43 mg/dL over 6 months. After switching to dasatinib, the creatinine level decreased from 1.43 to 1.1 mg/dL. The other patient had gradual elevation in her creatinine level from 1.6 to 3.75 mg/dL, likely due to hypertensive nephropathy, she switched to nilotinib, and her creatinine stabilized thereafter.

**Table 2:** Reported treatment‐emerged adverse events

Total N = 38	Number (%)		
	Original imatinib	Generic imatinib Persistent symptoms	Generic imatinib New or worsening symptoms
Non hematologic AE (any grade)			
Muscle cramps	22 (58)	11 (29)	8 (21)
Edema	21 (55)	7 (18)	5 (13)
Diarrhea	14 (37)	0 (0)	6 (16)
Fatigue	13 (34)	5 (13)	5 (13)
Nausea	11 (29)	4 (11)	7 (18)
Increased creatinine	5 (13)	2 (5)	4 (11)
Rash	4 (11)	0 (0)	1 (3)
Weight gain	3 (8)	0 (0)	0 (0)
Increased LDH	3 (8)	2 (5)	1 (3)
Increased Bilirubin	1 (3)	0 (0)	0 (0)
Increased ALT	1 (3)	1 (3)	0 (0)
Insomnia	1 (3)	0 (0)	2 (5)
Pruritus	1 (3)	0 (0)	0 (0)
Hematologic AE (any grade)			
Anemia	13 (34)	6 (16)	2 (5)
Thrombocytopenia	3 (8)	1 (3)	0 (0)
Neutropenia	1 (3)	0 (0)	2 (5)

## 4 DISCUSSION

Our observational study is the first report describing the efficacy and safety outcomes of generic imatinib in the United States after its approval by the FDA and entry into the market. FDA regulations for generic agents require bioequivalence studies demonstrating that the rate and extent of drug absorption fall within 80%‐125% of those of the original drug.^20^ Post‐marketing pharmacovigilance by reporting adverse events to the FDA is also encouraged. Many reports questioned the true bioequivalence of generic form of imatinib, mainly because of structural difference in the active ingredients. The generic form of imatinib has a different crystal form (alpha crystal form) than the branded imatinib (beta crystal form), which is less stable at room temperature. However, this polymorphism did not affect the solubility and bioavailability of the product, and it is generally considered clinically insignificant.^21^ Many bioequivalence studies were conducted in healthy volunteers and patients. In 30 healthy male South American volunteers, both original and generic forms of 400 mg imatinib have similar mean AUC and Cmax, with a comparable adverse event profile.^22^ Another multicentric randomized crossover bioequivalence study was done on 42 patients diagnosed with CML comparing pharmacokinetics, including Tmax, Cmax and AUC 0‐24 for both original and generic forms of imatinib. Sun Pharma‐Ranbaxy Lab Limited manufactured the generic form in India. Both formulations have the same rate and extent of absorption, and same safety profile, consistent with the FDA bioequivalence requirements.^23^ Similar bioequivalence studies were done with other generic forms, including Neopax and Imakrebin.^24^

Several manufacturers have been producing and distributing generic forms in developing countries before patent expiration. In most instances, substandard pharmaceuticals are distributed to those countries without inspection and strict oversight.^20^ Many reports have been published from those countries claiming the failure of generic forms of imatinib, mainly using CIPLA Imatib manufactured in India. Four cases reported separately from Egypt described patients who lost their complete hematologic responses (CHR) when they switched to generic imatinib and later responded again to the original imatinib.^14^, ^15^, ^18^ Another case report from Morocco described one patient with hematologic relapse with generic imatinib (Imatinib‐COPER), and then achieved CHR again on original imatinib.^16^ In a prospective study from Iraq, 126 patients switched from branded to generic imatinib (CIPLA Imatib, India) for at least 9 months described a loss of CHR in 25% by 6 months. Patients were then switched back to the original form and had an increase in CHR rate by 10%. In addition, the generic imatinib was not well tolerated with the most commonly reported adverse events, including bone pain in 87.3% of patients, muscle cramps in 81.7%, fluid retention in 67.5%, and nausea in 52.4% of patients.^17^ In Colombia, twelve patients switched to second‐generation TKI because of resistance and intolerance to generic imatinib.^19^

In our retrospective study, none of our patients lost MMR while on generic imatinib. This is an indirect suggestion that a well‐regulated and quality‐controlled generic imatinib can maintain the same efficacy of its original form. Many trials evaluated switching from original to generic imatinib and reported no difference in efficacy and safety between the two forms.^25^, ^26^, ^27^, ^28^ Kang et al evaluated 30 patients in Canada who switched to generic imatinib (Apotex‐TEVA) and reported loss of MMR in one patient and loss of CHR in one patient with all other patients maintaining their response. There were no significant differences reported in the safety profile of the generic imatinib during a follow‐up period of 12 months.^29^ Another observational study in Italy on 294 patients who were treated with original imatinib for at least 6 months, then switched to generic imatinib (Imatinib, Sandoz) showed stabilization, improvement and worsening of molecular responses in 61%, 25% and 14% of patients, respectively.^28^

One of the limitations of our study is that most of our patients (74%) were in MR4.5 with undetectable transcripts (≥100 000 ABL copies) for a median of 9 years before switching to generic imatinib. In our experience, the risk of MMR loss after discontinuation of imatinib after 6 years of continuous CMR is only approximately 7%.^30^ However, in this report, three of five patients who electively discontinued generic imatinib had a molecular relapse after a median follow‐up of 12 months.^31^, ^32^ Thus, the generic formulation at least in those patients demonstrated a clinical efficacy that was lost after discontinuation. It is also encouraging that most patients with detectable transcripts had some level, even if modest, of decrease in transcript levels. Another limitation is the lack of information regarding the type of generic imatinib used as this information was not always known to the patients and to the management team.

Moreover, our study did not include patients who received generic imatinib as the initial therapy for their CML. Reports from Turkey, Algeria, and Bosnia published on outcomes of patients using generic imatinib as initial therapy show conflicting results. In a retrospective comparative study in Turkey, patients using generic imatinib (Imatis, Imavec, Imatenil) in the upfront setting had similar responses in regards to CHR, CCyR, and MMR compared to those who received original imatinib. They also had similar early molecular responses defined as *BCR‐ABL* PCR less than 10% at 3 months to those who receive original imatinib in the upfront setting, which correlates with better event‐free survival.^33^, ^34^ In Algeria, Entasoltan et al retrospectively reviewed 355 patients newly diagnosed with CML and treated with upfront generic imatinib (CIPLA Imatib, India). At a median follow‐up of 46 months, 83% of patients achieved CHR at 3 months, 35% achieved MMR at one year and 67% at 2 years; of them, 34% of patients were in CMR. They also concluded that the drug was safe, with just 8% of patients switching to second‐generation TKI because of intolerance.^35^ Razmkhah et al^36^ reported on 30 patients treated with generic imatinib (CIPLA Imatib, India) as first line therapy for CML chronic phase, and had CHR and CMR in 90% and 46.7%, respectively. Awidi et al conducted an observational, multicentric prospective study on 91 patients receiving generic imatinib (Cemivil, Hikma Pharmaceuticals) either in upfront setting or after switch from original imatinib. Their study indicated the same efficacy and safety in both populations, with 85% of patients achieving CHR at 3 months and 45% of patients achieved MMR at one year, also 85% of adverse events were mild.^26^ Although these rates may seem lower than expected compared to the literature from larger studies, there are many variables (patient characteristics, availability of frequent monitoring, etc) that may be different in these settings compared to randomized and even observational studies from other parts of the world. In contrast, generic imatinib (Anzovip, Meaxin, Plivatinib) used in frontline treatment of CML in 27 patients from Bosnia and Herzegovina was suboptimal; at 3 years, 81% of patients achieved a complete cytogenetic response, and 52% of patient switched to nilotinib due to either treatment failure or adverse events.^37^ Our results, albeit observational in nature, suggest that a switch from original imatinib to generic imatinib maintains efficacy and preserves safety. These results are reflective of the generic formulations available in the US and it is possible that they might not be extrapolated to other generic formulations including substitutes and copies with lesser quality controls and regulatory oversight. Close monitoring of patients is required in all instances of patients receiving imatinib, whether generic or original, for optimal care.

## CONFLICT OF INTEREST

JC has research support from Ariad, Bristol‐Myers‐Squibb (BMS), Novartis, Pfizer, Sun Pharma, and Teva, and is a consultant for Ariad, BMS, Fusion Therapeutics, Pfizer, and Teva.
